# Efficacy and Safety of Ashwagandha (Withania somnifera) Root Extract for Improvement of Sexual Health in Healthy Women: A Prospective, Randomized, Placebo-Controlled Study

**DOI:** 10.7759/cureus.30787

**Published:** 2022-10-28

**Authors:** Ashutosh Ajgaonkar, Mukta Jain, Khokan Debnath

**Affiliations:** 1 Obstetrics and Gynecology, Vedanta Hospital, Mumbai, IND; 2 Pharmacology, D.Y. Patil University School of Medicine, Navi Mumbai, IND; 3 Family Medicine, Prakruti Hospital, Mumbai, IND

**Keywords:** ghq-28, satisfying sexual encounters, fsds, fsfi, female sexual function, ashwagandha

## Abstract

Background

Poor sexual function is a widespread problem affecting about 40% of women and this may worsen their quality of life. Ashwagandha (*Withania somnifera*) an adaptogenic herb has been reported to improve sexual satisfaction, sleep, and quality of life in women.

Objective

The purpose of the study was to evaluate the efficacy and safety of standardized Ashwagandha root extract in improving sexual function in healthy females.

Methods

In this prospective, randomized, placebo-controlled study, 80 women between 18 and 50 years of age without any hormonal disturbances and having hypoactive sexual desire disorder (HSDD) with a Female Sexual Function Index (FSFI) score <26, or Female Sexual Distress Scale (FSDS) score >11 were randomized to receive either capsule containing standardized Ashwagandha root extract 300mg twice daily (n=40), or identical placebo (n=40) for eight weeks. Sexual function was assessed using FSFI, FSDS, and Satisfying Sexual Encounters (SSEs). Assessments were done at baseline, four weeks, and eight weeks. Quality of life (QoL) was assessed using the general health questionnaire (GHQ-28) scale, and safety was assessed using clinical signs and symptoms. Repeat measures analysis of variance (ANOVA) was used for the assessment of treatment effect at different time periods. Nominal data were analyzed for differences using Fischer’s Chi-square test.

Results

There was statistically significant improvement (p<0.0001) in FSFI scores with Ashwagandha [14.20 (0.98) at baseline to 22.62 (2.06) at week 8] as compared to placebo [14.17 (0.71) at baseline to 19.25 (2.23) at eight weeks], and this improvement was observed in all sub-scales (desire, arousal, lubrication, orgasm, sexual satisfaction, and pain) of the FSFI scale. There was a greater improvement (p<0.0001) in FSDS scores with AG as compared to placebo. Although not statistically significant (p, 0.078), there was a greater reduction (improvement) in GHQ-28 scores at eight weeks with Ashwagandha as compared to placebo, and this trend was observed for all domains of GHQ-28 (global, physical, psychological, and social function). More women with Ashwagandha had improvement in SSEs at week 4 (p, 0.017) and week 8 (p, 0.002) as compared to placebo. Adverse events were comparable in the two groups. Two women reported nausea and one reported drowsiness with AG, whereas two reported nausea, one reported drowsiness and one reported nausea with drowsiness in the placebo group.

Conclusions

Oral administration of Ashwagandha 300mg twice daily administered for eight weeks improves the female sexual health in otherwise healthy women who do not have any hormonal disturbances. Ashwagandha is a known adaptogen, maintains general well-being and improves vitality.

## Introduction

Sexual function is an essential component of life and poor sexual function may worsen the quality of life in women. Female sexual dysfunction (FSD) is a prevalent problem, affecting about 40% of women [1]. Women with sexual dysfunction may have female sexual arousal disorder (FSAD), Female Orgasmic Disorder (FOD), or Hypoactive Sexual Desire Disorder (HSDD). Some women may have combined genital and subjective arousal disorders or isolated genital sexual arousal disorders [2]. Several factors including medical and physical conditions like heart disease, diabetes, thyroid disease, neurological, and even simple fatigue could be the plausible causes for FSD [3]. In addition, psychological and emotional issues like depression, stress, and anxiety states could contribute significantly to FSD.

Ashwagandha (Withania somnifera [WS] fam. Solanaceae) commonly known as “Indian Winter cherry” is one of the most important herbs in Ayurveda (the traditional system of medicine in India) used for millennia as a Rasayana for its wide-ranging health benefits [4]. Ashwagandha is found across India and south Asia and is cultivated in the dry western parts of India. The important phytochemicals in Ashwagandha which are the active constituents responsible for the therapeutic efficacies of the plant are the withanolides which are steroidal lactones. Ashwagandha is considered an “adaptogen” as it protects the body from stress and helps the body to recover from the effects of stress. It reduces serum cortisol levels in patients with chronic stress, restores adrenal functions and normalizes the sympathetic nervous system [5]. Ashwagandha improves memory and promotes a healthy sexual and reproductive balance. It helps in improving cell-mediated immunity with its potent antioxidant properties. It is an adaptogenic botanical grown in India that is revered for its ability to balance, energize, rejuvenate, and revitalize [6]. The chemical profile of several extracts and formulations of WS has been well documented in previous studies, and the extracts contain a variety of phytochemicals that makes it a strong therapeutic agent [7]. WS has numerous physiological actions including anti-inflammatory, neuroprotective, adaptogen, cognitive-enhancing effects and normalizing of the sleep cycle. Extract of Ashwagandha root helps manage stress by reducing the stress hormone levels in the blood. This stress-relieving effect of Ashwagandha may have a direct contribution towards better sexual health. Ashwagandha can improve energy, stamina, and endurance. Fatigue is very often cited as an inhibiting factor in reduced sexual activity, and stress is associated with an increase in serum cortisol which can be associated with gonadal and sexual dysfunction [8]. Consumption of extract of Ashwagandha root may reduce FSD in women with androgen deficiency syndrome, which is seen as contributing factor towards a lack of sexual desire [9]. Also, animal studies have confirmed Ashwagandha's influence on sex hormone production, as demonstrated by its effects on luteinizing hormone, follicle-stimulating hormone, testosterone, and progesterone [10,11]. Thus, regular consumption of Ashwagandha has the potential to improve sexual function and improve the quality of life in women.

## Materials and methods

Study design

This 8-week prospective, randomized, double-blind, placebo-controlled clinical study was conducted to evaluate the efficacy and safety of oral administration of standardized Ashwagandha root extract in improving sexual health in healthy women who do not have any hormonal disturbances.

The study was conducted in accordance with the Helsinki Declaration (1989 amendment) and the study protocol was approved by the Institutional Ethics Committee of DY Patil Medical College and Hospital, Navi Mumbai (Ref. #: DYP/IEC/2019-08-158, dated 12 December 2019). The Consolidated Standards of Reporting Trials (CONSORT) guidelines for designing and reporting controlled trials were followed in the conduct and reporting of this study. Written informed consent was obtained from all participants prior to the enrolment. Each participant was explained in detail about the study objective and the expected outcome before taking the consent.

Sample size

The sample size was based on the results of previously reported data for reduction in FSDS with eight weeks of ashwagandha. [9] A sample size of seven in each group provides 90% power to detect a difference of -5.6 for change in FSDS score after eight weeks between the AG and PL with a significance level (alpha) of 0.050 using a one-sided two-sample t-test. However, we planned to include 100 women in the study with 50 in each group.

Study participants

Married or unmarried healthy women between 18 and 50 years of age attending the study site were screened for study eligibility based on the study inclusion and exclusion criteria. Women having regular menstrual periods were inquired about any signs and symptoms suggestive of HSDD. Those who had HSDD as per the Diagnostic and Statistical Manual of Mental Disorders (DSM)-IV Sexual Desire Screening Questionnaire were included in the study. HSDD is defined as “Persistently or recurrently deficient (or absent) sexual fantasies and desire for sexual activity” along with “marked distress or interpersonal difficulty.” Women who had a Female Sexual Function Index (FSFI) total score of <26 and/or a Female Sexual Distress Scale (FSDS) score of >11 at screening were enrolled. All women and their partners were willing to try to have regular sexual intercourse activity (four or more attempts in four weeks) and were able and willing to maintain a diary of sexual events. Women were in a stable, monogamous, heterosexual relationship that was secure and communicative, for at least one year prior to the screening. Participating women's partner was expected to be physically present at their place of stay for a minimum of 50% of the study period. All couples used a medically acceptable method of contraception for at least three months prior to screening and continue to use that during the study period.

Women having any acute illness which may hamper the study participation, and those with any clinically significant medical history, medical finding or an on-going medical or psychiatric condition were excluded. Baseline serum hormones were estimated as a screening procedure in all women. Women with any deviation from the normal serum levels for estrogen, progesterone and testosterone were excluded from participation. Women with any other situation which in the opinion of the investigator could jeopardize their safety, impact the validity of the study results or interfere with study completion were excluded. Those who participated in other studies, including macro/micro/any other forms of dietary supplements/multivitamins or disease-specific oral nutrition supplements (for improving sexual function) during the three months prior to screening were not included. Women with a known history of hypersensitivity reactions, or a history of alcohol or substance abuse within the past year were excluded. Women who indicated that their sexual partner has inadequately treated sexual problems that could interfere with the response to treatment were also excluded. Those within the menopausal transition or menopause or who have had a hysterectomy were excluded.

Randomization and blinding

Enrolled women were randomly assigned to Ashwagandha (AG) or Placebo (PL) in a 1:1 randomization ratio. Randomization was performed using a computer-generated pre-determined randomization list (Rando version 1.2, Windows). The AG and PL products were manufactured and packed in identical containers and labeled equivalently to ensure blinding. The investigator received the randomization codes in separate envelopes for each study participant and was instructed to open the envelope only after assigning the study number to the eligible participant. An independent investigator who was blinded collected the outcome measures at each scheduled visit.

Interventions

Women in the AG group received a capsule containing standardized Ashwagandha root extract (Ixoreal Biomed, LA, CA) 300mg, whereas women in the PL group received a capsule identical to AG containing 300mg starch. Both treatments were administered orally two times daily with water for a period of eight weeks. The sexual partners of the participating women were not provided with any study-specific intervention.

Efficacy assessments

All women underwent a detailed medical and surgical history and were subjected to detailed clinical examination for any physical signs/symptoms, and vital parameters at baseline and all follow-up visits. The primary efficacy outcomes were the mean improvement in FSFI scores and FSDS scores from baseline to the end of four and eight weeks. Secondary efficacy outcomes were: i) mean change in the quality of life assessed using the GHQ-28 questionnaire from baseline to end of eight weeks; ii) improvement in the total sexual encounters and satisfying sexual encounter (SSE) computed as mean change in SSEs from baseline to end of four and eight weeks, and iii) proportion of patients reporting improvement in SSEs at four and eight weeks. 

FSFI scale is a 19-item self-reported (patient) survey instrument developed by Rosen et al. [12] assessed the extent of sexual dysfunction in participating women. The instrument has sub-scores for different domains for desire, arousal, lubrication, orgasm, satisfaction, and pain. The FSFI total score is a weighted sum of these. FSDS is a self-report 12-item survey instrument [13] that measures sexually related personal distress in women. The items correspond to different dimensions of sexual distress and were rated based on the occurrence during the previous 30 days. A record of all sexual activity was maintained to collect data for “total sexual encounters” and “satisfying sexual encounters.” The FSFI, FSDS and sexual activity records were recorded at baseline, at four weeks, and the study termination (eight weeks). The 28-item General Health Questionnaire (GHQ-28) was used to assess the quality of life [14]. The GHQ-28 has four sub-scales which include: i) physical symptoms (1-7), ii) anxiety symptoms (8-14), iii) social function (15-21), and iv) depression symptoms (22-28). Each sub-scale had seven items with each question scored on a Likert scale (0-3). The total scores range from 0 to 84, with lower scores indicating a better quality of life. The 28-question form had the advantage of being useful for all the members of the society [15].

Safety assessments

The safety outcomes were clinical safety recorded as the number and proportion of Treatment Emergent Adverse Events (TEAEs) and Treatment Emergent Serious Adverse Events (TESAE) during the study period.

Compliance assessment

Compliance was assessed using the tablet count, and the patient was considered compliant if more than 80% of the medication was consumed as per the protocol.

Statistical methods

The study sample size was determined based on the improvement in total FSFI scores reported by Dongre et al. [9]. The mean (SD) reduction in FSFI total scores at the end of eight weeks of treatment with Ashwagandha and placebo were 10.29 (1.75) and 6.43 (1.89), respectively [9]. Group sample sizes of seven (AG) and seven (PL) achieve 90% power to detect a score difference of 3.9 for FSFI between AG and PL at a significance level (alpha) of 0.050 using a two-sided two-sample t-test. However, we planned to include 80 women in the study with 40 in each of the two treatment groups.

All statistical computations were done on the windows-based program MedCalc® Statistical Software version 20.015 (MedCalc Software Ltd, Ostend, Belgium; https://www.medcalc.org; 2021). Efficacy analysis was performed on the per-protocol (PP) data (n = 72), whereas safety analysis was performed on the intent-to-treat (ITT) data (n = 80). Summary statistics for all the parameters were performed and the results were presented as means with a 95% confidence interval (C.I.) for continuous data and counts with percentages for categorical data. Categorical and nominal data were analyzed for differences between the two groups using the chi-square test (Fisher’s exact test). For measurement data, repeat measures analysis of variance (ANOVA) was used for the assessment of treatment effect at different time periods, with Dunnett’s test for between group comparisons. All analyses were done using two-sided tests at alpha 0.05 (95% confidence levels), and a p-value of <0.05 was the cut-off for rejecting the null hypothesis.

## Results

Demography and baseline data

**Figure 1:**
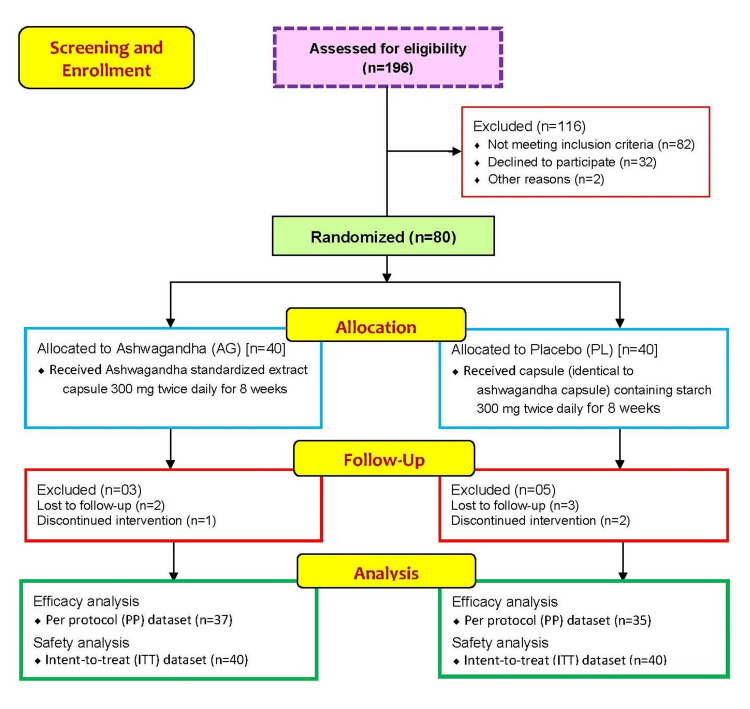
CONSORT flow representation of the patient enrollment, allocation, follow-up and analysis for this study CONSORT: Consolidated Standards Of Reporting Trials

A total of 196 participants were screened and assessed for eligibility initially, of which, 116 failed the eligibility criteria (Figure 1). The remaining 80 participants underwent randomization and were allocated to receive either capsule containing Ashwagandha standardized root extract 300mg twice daily (n=40), or an identical placebo (n=40) in a 1:1 ratio. Three participants from the Ashwagandha group and five from the placebo group were excluded due to follow-up loss or failure of medication adherence. Compliance was poor in one patient from AG and two patients from the PL group, and data of these women was excluded from efficacy analyses. Poor compliance was not due to adverse events. The final efficacy analyses were done on the per-protocol (PP) dataset of seventy-two women (37 in AG and 35 in the PL group), whereas safety analyses were done on the intent-to-treat (ITT) dataset of all eighty women.

Table 1 shows the demography and baseline data for patients enrolled and randomized (ITT dataset). The two groups were comparable at baseline with respect to the demography and baseline scores.

**Table 1:** Demography and baseline data for all parameters in the ITT dataset SBP: systolic blood pressure; DBP: diastolic blood pressure; FSFI: female sexual function index; FSDS: female sexual distress scale; GHQ-28: general health questionnaire-28; ITT: intent-to-treat; CI: confidence intervals

ITT dataset	Ashwagandha (AG, n=40)		Placebo (PL, n=40)		t-test		Mean Diff.	95% C.I. for diff.
	Mean	SD	Mean	SD	‘t’	‘p’		
Age (yrs.)	29.10	5.69	29.85	6.15	-0.566	0.573	-0.75	-3.39 to 1.89
Height (cm)	147.10	7.96	148.58	9.04	-0.775	0.441	-1.47	-5.27 to 2.32
Weight (Kg)	69.83	9.25	68.85	11.04	0.428	0.670	0.98	-3.56 to 5.51
BMI (Kg/sq.m)	32.33	4.22	31.26	4.77	1.059	0.293	1.07	-0.94 to 3.07
SBP (mm Hg)	119.35	11.25	117.00	15.06	0.791	0.432	2.35	-3.57 to 8.27
DBP (mm Hg)	77.30	4.83	76.50	8.34	0.525	0.601	0.80	-2.23 to 3.83
Pulse rate (/min.)	72.05	2.29	70.70	2.24	2.665	0.009	1.35	0.34 to 2.36
Temperature (0F)	98.19	0.11	98.19	0.17	-0.233	0.817	-0.01	-0.07 to 0.06
Respiratory rate (per min.)	17.05	1.50	17.35	1.59	-0.866	0.389	-0.30	-0.99 to 0.39
FSFI scale								
Desire	2.04	0.57	2.08	0.54	-0.282	0.779	-0.04	-0.28 to 0.21
Arousal	2.36	0.38	2.29	0.30	0.916	0.362	0.07	-0.08 to 0.22
Lubrication	2.45	0.49	2.40	0.40	0.450	0.654	0.05	-0.15 to 0.24
Orgasm	1.92	0.39	1.93	0.36	-0.118	0.906	-0.01	-0.18 to 0.16
Satisfaction	2.44	0.45	2.40	0.38	0.453	0.652	0.04	-0.14 to 0.23
Pain	3.05	0.49	3.06	0.53	-0.066	0.948	-0.01	-0.23 to 0.22
Total FSFI Score	14.26	1.02	14.16	0.73	0.530	0.598	0.10	-0.29 to 0.50
FSDS								
FSDS score	17.55	3.40	17.53	3.07	0.733	0.466	0.10	-0.17 to 0.37
Sexual encounters								
Total encounters	4.40	1.71	4.33	2.08	0.176	0.861	0.08	-0.77 to 0.92
Successful encounters	1.75	0.90	1.98	1.25	-0.924	0.358	-0.23	-0.71 to 0.26
GHQ-28 scale								
Total score	52.95	11.96	52.03	10.80	0.363	0.718	0.93	-4.15 to 6.00
Physical score	14.05	2.89	12.28	3.34	2.540	0.013	1.78	0.38 to 3.17
Anxiety score	12.25	4.11	11.58	3.85	0.758	0.451	0.68	-1.10 to 2.45
Social function score	13.75	3.15	14.10	2.59	-0.543	0.589	-0.35	-1.63 to 0.93
Depression score	12.90	3.40	14.08	2.63	-1.727	0.088	-1.18	-2.53 to 0.18

Female sexual function index (FSFI)

**Figure 2:**
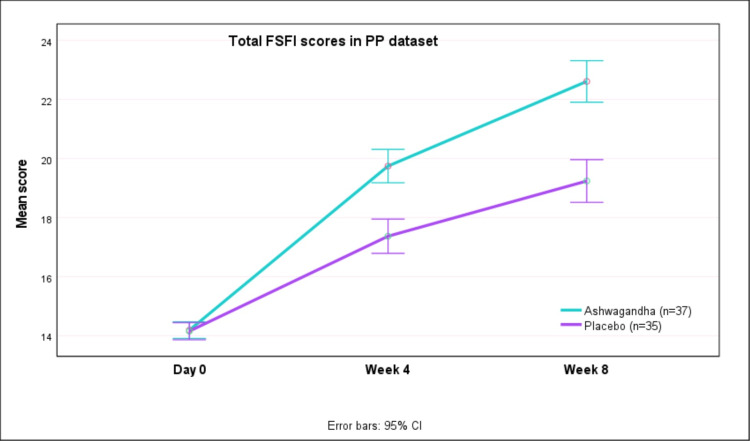
Total FSFI scores on day 0, week 4 and week 8 in PP dataset FSFI: female sexual function index; PP: per-protocol; CI: confidence interval

Table 2 presents the FSFI (total and sub-scale) scores at baseline and the change in scores at week 4 and week 8 after therapy in the PP dataset. Greater improvements in FSFI scores were seen with AG as compared to PL (p<0.0001). The mean (SD) FSFI total scores increased from 14.20 (0.98) at baseline to 22.62 (2.06) at week 8 with AG, whereas with PL the scores increased from 14.17 (0.71) at baseline to 19.25 (2.23) after eight weeks (Figure 2). Thus, there was greater improvement in FSFI scores with AG as compared to placebo (p<0.0001). This improvement was observed in all sub-scales (desire, arousal, lubrication, orgasm, sexual satisfaction, and pain) of the FSFI scale.

**Table 2:** FSFI scale scores at baseline and during study period in PP dataset FSFI: female sexual function index; PP: per-protocol; CI: confidence interval

	Ashwagandha (AG, n=37)		Placebo (PL, n=35)		t-test		Mean Difference	95% C.I. for diff.	Effect size (Cohen’s)	
	Mean	SD	Mean	SD	t'	p'			‘d'	95% C.I. for ‘d’
Desire sub-scale										
Day 0	2.01	0.56	2.07	0.54	-0.445	0.657	-0.06	-0.32 to 0.20	-0.11	-0.57 to 0.36
Change at Week 4	0.68	0.54	0.55	0.59	0.954	0.344	0.13	-0.14 to 0.39	0.22	-0.24 to 0.69
Change at Week 8	1.44	0.59	1.25	0.79	1.204	0.233	0.20	-0.13 to 0.52	0.28	-0.18 to 0.75
Arousal sub-scale										
Day 0	2.35	0.36	2.30	0.30	0.660	0.511	0.05	-0.10 to 0.21	0.16	-0.31 to 0.62
Change at Week 4	0.57	0.38	0.36	0.23	2.832	0.006	0.21	0.06 to 0.36	0.67	0.19 to 1.14
Change at Week 8	0.94	0.48	0.34	0.51	5.236	<0.0001	0.61	0.38 to 0.84	1.23	0.73 to 1.74
Lubrication sub-scale										
Day 0	2.46	0.50	2.43	0.42	0.206	0.838	0.02	-0.20 to 0.24	0.05	-0.41 to 0.51
Change at Week 4	0.96	0.53	0.54	0.58	3.210	0.002	0.42	0.16 to 0.68	0.76	0.28 to 1.23
Change at Week 8	1.20	0.61	0.69	0.50	3.892	<0.0001	0.51	0.25 to 0.77	0.92	0.43 to 1.40
Orgasm sub-scale										
Day 0	1.90	0.36	1.95	0.36	-0.668	0.507	-0.06	-0.23 to 0.11	-0.16	-0.62 to 0.31
Change at Week 4	1.37	0.68	0.77	0.49	4.236	<0.0001	0.60	0.32 to 0.88	1.00	0.50 to 1.49
Change at Week 8	1.44	0.63	0.87	0.46	4.371	<0.0001	0.57	0.31 to 0.84	1.03	0.53 to 1.52
Satisfaction sub-scale										
Day 0	2.44	0.47	2.39	0.38	0.462	0.645	0.05	-0.15 to 0.25	0.11	-0.35 to 0.57
Change at Week 4	0.78	0.60	0.27	0.50	3.953	<0.0001	0.52	0.26 to 0.78	0.93	0.44 to 1.42
Change at Week 8	1.24	0.71	0.30	0.68	5.778	<0.0001	0.95	0.62 to 1.27	1.36	0.84 to 1.87
Pain sub-scale										
Day 0	3.05	0.48	3.02	0.53	0.240	0.811	0.03	-0.21 to 0.27	0.06	-0.41 to 0.52
Change at Week 4	1.19	0.83	0.73	0.80	2.448	0.017	0.47	0.09 to 0.85	0.58	0.10 to 1.05
Change at Week 8	2.15	0.82	1.65	0.76	2.691	0.009	0.50	0.13 to 0.87	0.63	0.16 to 1.11
Total FSFI Score										
Day 0	14.20	0.98	14.17	0.71	0.169	0.866	0.03	-0.37 to 0.44	0.04	-0.42 to 0.50
Change at Week 4	5.55	1.90	3.21	1.76	5.404	<0.0001	2.34	1.48 to 3.20	1.27	0.76 to 1.78
Change at Week 8	8.42	1.96	5.08	2.05	7.061	<0.0001	3.34	2.39 to 4.28	1.67	1.12 to 2.20

 

FSDS and SSEs

**Figure 3:**
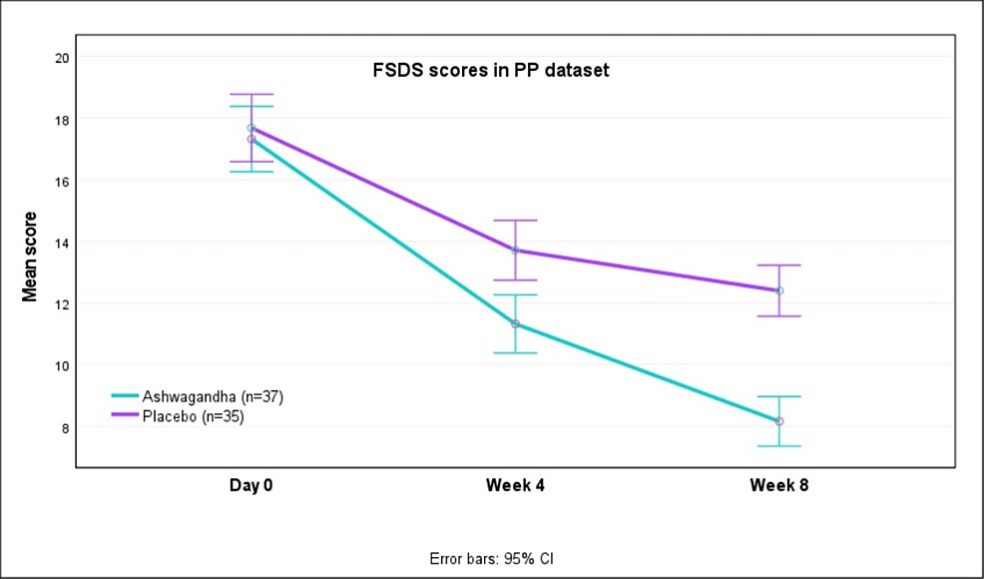
FSDS scores on day 0, week 4 and week 8 in PP dataset FSDS: female sexual distress scale; PP: per-protocol; CI: confidence interval

Table 3 presents the FSDS and sexual encounters at baseline and after week 4/week 8 of therapy in the PP dataset. Greater reductions in FSDS scores were seen with AG as compared to PL (p<0.0001). Also, the mean number of SSEs increased significantly with AG as compared to PL (p<0.0001). The mean (SD) total FSDS scores decreased from 17.32 (3.38) at baseline to 8.16 (2.36) at week 8 with AG, whereas with PL the scores decreased from 17.69 (3.08) at baseline to 12.40 (2.55) after eight weeks (Figure 3). Thus, there was greater improvement in FSDS scores with AG as compared to PL (p<0.0001).

**Table 3:** FSDS and number of sexual encounters at baseline and during study period in PP dataset. FSDS: female sexual distress scale; PP: per-protocol; CI: confidence interval

	Ashwagandha (AG, n=37)		Placebo (PL, n=35)		t-test		Mean Difference	95% C.I. for diff.	Effect size (Cohen’s)	
	Mean	SD	Mean	SD	t'	p'			‘d'	95% C.I. for ‘d’
FSDS scale										
Day 0	17.32	3.38	17.69	3.08	-0.473	0.638	-0.36	-1.89 to 1.16	-0.11	-0.57 to 0.35
Change at Week 4	-6.00	3.11	-3.97	2.73	-2.936	0.004	-2.03	-3.41 to -0.65	-0.69	-1.17 to -0.21
Change at Week 8	-9.16	2.91	-5.29	3.21	-5.370	<0.0001	-3.88	-5.32 to -2.44	-1.27	-1.77 to -0.75
Total sexual encounters										
Day 0	4.41	1.77	4.17	2.04	0.521	0.604	0.23	-0.66 to 1.13	0.12	-0.34 to 0.59
Change at Week 4	0.78	1.18	0.49	1.80	0.833	0.407	0.30	-0.42 to 1.01	0.197	-0.27 to 0.66
Change at Week 8	0.81	1.78	0.06	2.11	1.641	0.105	0.75	-0.16 to 1.67	0.387	-0.08 to 0.85
Successful encounters										
Day 0	1.76	0.93	1.89	1.21	-0.510	0.611	-0.13	-0.63 to 0.37	-0.12	-0.58 to 0.34
Change at Week 4	1.70	0.97	1.03	1.27	2.540	0.013	0.67	0.14 to 1.20	0.599	0.12 to 1.07
Change at Week 8	2.32	1.08	1.11	1.08	4.752	<0.0001	1.21	0.70 to 1.72	1.120	0.62 to 1.61

 

**Figure 4:**
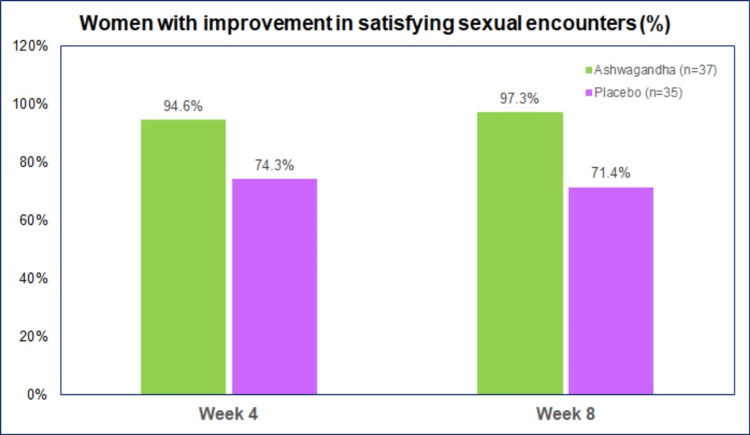
Women with improvement in SSEs at week 4 and week 8 SSE: satisfying sexual encounters

There was an improvement in the number of women reporting “satisfying sexual encounters” with both AG and PL (Figure 4). However, more women with Ashwagandha had improvement in SSEs than placebo both at week 4 (p, 0.017) and week 8 (p, 0.002). A decrease in SSEs was observed in two (5.4%) women on AG and seven (20.0%) on PL after four weeks, and three (8.6%) women on PL after eight weeks. There were no women with decreased SSEs after eight weeks.

General health questionnaire (GHQ-28)

**Figure 5:**
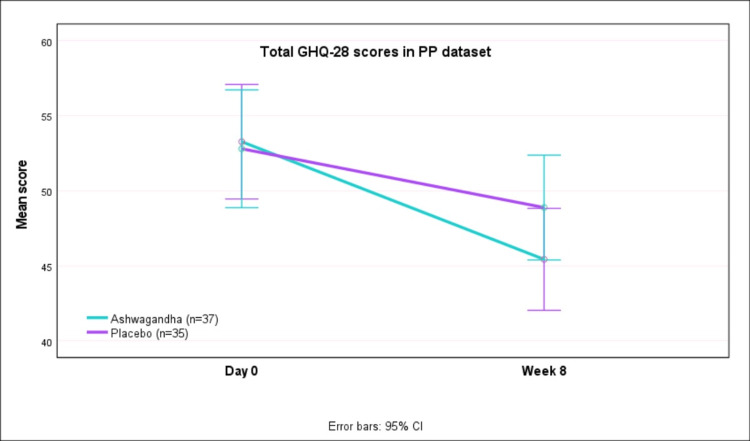
GHQ-28 total scores at day 0 and week 8 in PP dataset GHQ-28: general health questionnaire-28; PP: per=protocol; CI: confidence interval

Table 4 presents the GHQ-28 scores at baseline and after week 8 in the PP datasA greaterater reduction in scores was seen with AG as compared to PL (p<0.05). The mean (SD) GHQ-28 scores decreased from 53.27 (12.25) at baseline to 45.43 (11.90) at week 8 with AG, whereas with PL the scores decreased from 52.80 (10.94) at baseline to 48.89 (8.43) after eight weeks (Figure 5). Although not statistically significant (p, 0.078), there was a greater reduction (improvement) in GHQ-28 scores with AG as compared to PL. This trend was seen for all domains of GHQ-28 (global, physical, psychological and social function).

**Table 4:** GHQ-28 scores at baseline and during study period in PP dataset GHQ-28: general health questionnaire-28; PP: per-protocol; CI: confidence interval

	Ashwagandha (AG, n=37)		Placebo (PL, n=35)		t-test		Mean Difference		Effect size (Cohen’s)	
	Mean	SD	Mean	SD	t'	p'		95% C.I. for diff.	‘d'	95% C.I. for ‘d’
Total GHQ-28 score										
Day 0	53.27	12.25	52.80	10.94	0.171	0.864	0.47	-5.00 to 5.94	0.04	-0.42 to 0.50
Change at Week 8	-7.84	8.74	-3.91	9.87	-1.789	0.078	-3.92	-8.30 to 0.45	-0.42	-0.89 to 0.05
Physical score										
Day 0	14.14	2.93	12.60	3.37	2.067	0.042	1.54	0.05 to 3.02	0.49	0.02 to 0.95
Change at Week 8	-1.78	3.00	-0.74	3.04	-1.461	0.148	-1.04	-2.46 to 0.38	-0.34	-0.81 to 0.12
Anxiety score										
Day 0	12.35	4.18	11.77	3.93	0.606	0.546	0.58	-1.33 to 2.49	0.14	-0.32 to 0.61
Change at Week 8	-2.95	3.83	-1.54	5.77	-1.222	0.226	-1.40	-3.69 to 0.89	-0.29	-0.75 to 0.18
Social function score										
Day 0	13.92	3.17	14.29	2.61	-0.535	0.594	-0.37	-1.73 to 1.00	-0.13	-0.59 to 0.34
Change at Week 8	-1.76	3.38	-0.83	3.02	-1.226	0.224	-0.93	-2.44 to 0.58	-0.29	-0.75 to 0.18
Depression score										
Day 0	12.86	3.54	14.14	2.59	-1.741	0.086	-1.28	-2.74 to 0.19	-0.41	-0.88 to 0.06
Change at Week 8	-1.35	3.15	-0.80	3.35	-0.720	0.474	-0.55	-2.08 to 0.98	-0.17	-0.63 to 0.29

 

Adverse events

Three women from each AG (7.5%) and PL (7.5%) group reported adverse events. Nausea was reported by two (5.0%) women from each AG and PL group, while drowsiness was reported by two (5.0%) women from the AG group and one (1.5%) from the PL group. One (1.5%) woman receiving AG reported both nausea and drowsiness. All events were of mild severity, were not associated with the study treatments, and were self-limiting.

Vital parameters

Table 5 presents the vital parameters at baseline (day 0) and after week 4 and week 8. No significant changes were observed in the vital parameters in the two groups (p>0.05).

**Table 5:** Vital parameters at baseline and during study period in PP dataset SBP: systolic blood pressure; DBP: diastolic blood pressure; PP: per-protocol; CI: confidence interval; ^0^F: degree fahrenheit

	Ashwagandha (AG, n=37)		Placebo (PL, n=35)		t-test		Mean Difference	95% C.I. for diff.	Effect size (Cohen’s)	
	Mean	SD	Mean	SD	t'	p'			‘d'	95% C.I. for ‘d’
SBP (mm Hg)										
Day 0	118.75	11.178	116.28	15.734	0.771	0.443	2.47	-3.92 to 8.86	0.18	-0.28 to 0.64
Week 4	118.27	9.106	117.14	9.570	0.512	0.610	1.13	-3.26 to 5.52	0.12	-0.34 to 0.58
Week 8	117.73	8.00	116.00	7.35	0.953	0.344	1.73	-1.89 to 5.35	0.22	-0.24 to 0.69
DBP (mm Hg)										
Day 0	77.29	4.90	76.00	7.746	0.854	0.396	1.30	-1.73 to 4.33	0.20	-0.26 to 0.66
Week 4	76.81	7.46	75.42	7.413	0.788	0.433	1.38	-2.12 to 4.88	0.19	-0.28 to 0.65
Week 8	76.16	4.76	74.57	6.10	1.235	0.221	1.59	-0.98 to 4.16	0.29	-0.17 to 0.75
Pulse rate (per min.)										
Day 0	72.05	2.37	70.45	2.17	2.968	0.004	1.60	0.52 to 2.67	0.70	0.22 to 1.17
Week 4	72.70	1.83	72.08	1.77	1.448	0.152	0.62	-0.23 to 1.47	0.34	-0.13 to 0.81
Week 8	72.54	2.60	72.51	1.83	0.049	0.961	0.03	-1.04 to 1.09	0.01	-0.45 to 0.47
Temperature (0F)										
Day 0	98.19	0.11	98.18	0.16	0.269	0.789	0.01	-0.06 to 0.08	0.06	-0.40 to 0.53
Week 4	98.25	0.13	98.23	0.21	0.468	0.642	0.02	-0.06 to 0.10	0.11	-0.35 to 0.57
Week 8	98.24	0.13	98.23	0.10	0.508	0.613	0.01	-0.04 to 0.07	0.12	-0.34 to 0.58
Respiratory rate (per min.)										
Day 0	17.13	1.53	17.25	1.46	-0.345	0.731	-0.12	-0.83 to 0.58	-0.08	-0.54 to 0.38
Week 4	18.54	1.46	18.00	1.18	1.714	0.091	0.54	-0.09 to 1.17	0.40	-0.06 to 0.87
Week 8	18.54	1.86	17.88	1.60	1.593	0.116	0.65	-0.16 to 1.47	0.38	-0.09 to 0.84

## Discussion

FSD is a multifaceted disorder, comprising anatomical, psychological, physiological, as well as social-interpersonal components. With several existing definitions for FSD, the most descriptive definition encompasses FSD as the persistent/recurring decrease in sexual desire or arousal, the difficulty/inability to achieve an orgasm, and/or the feeling of pain during sexual intercourse [16]. Currently, there are limited pharmacological options available for the treatment of FSD, which include psychoactive drugs (flibanserin and bremelanotide), transdermal testosterone, intravaginal dehydroepiandrosterone (DHEA), local estrogen therapy (LET) and ospemifene [17]. Ospemifene is a third-generation Selective Estrogen Receptor Modulator (SERM) reported to be useful in women with vulvovaginal atrophy, painful intercourse, vaginal dryness, and vulvar vestibular symptoms [18]. The newer agents aim at increasing blood flow to the genitals, improving androgen deficiencies and enhancing central nervous system stimulation. However, most of these pose safety issues [16]. Flibanserin, which is approved by the United States Food and Drug Administration (USFDA) for the treatment of low sexual desire in premenopausal women, causes potentially serious side effects like hypotension, sleepiness, nausea, fatigue, dizziness, and fainting, particularly if the drug is mixed with alcohol [16]. Another agent bremelanotide which is administered as an injection causes nausea, vomiting, flushing, headache, and local reaction at the injection site [16]. Estrogen therapy may lead to heart and blood vessel disease and cancer [19].

Sexual dysfunction is a frequent problem in women during pregnancy and counseling about healthy sexual function during pregnancy is required for most couples [20]. Women who underwent an operative vaginal delivery (n=132) had poor sexual function (lower orgasm scores, p<0.05) after six months compared to those with cesarean section delivery (n=92) and spontaneous vaginal delivery (n=45). Also, breastfeeding women were reported to have poor sexual function [21]. Poor sexual function is more common in women with rheumatoid arthritis (RA) and psoriatic arthritis (PsA), compared to healthy women [22], and female patients with RA and PsA should be screened for sexual dysfunction. Findings of a cross-sectional analysis of 3433 (2487 sexually active) women presenting for menopause or sexual health consult were reported from the Data Registry on Experiences of Aging, Menopause and Sexuality (DREAMS) by King et al. [23]. Of these, 75% had poor sleep quality, and 54% met the criteria for female sexual dysfunction. As reported by King et al., multivariable analysis shows that women with poor sleep quality were 1.48 times more likely to report female sexual dysfunction (95% C.I. 1.21 to 1.80, p < 0.001), and women with <5 hours sleep time had 63.3% prevalence of sexual dysfunction (p = 0.004). The authors also report that sexually active women were more likely to report good sleep quality compared with sexually inactive women (25.3% vs 20.5%, p = 0.003). The authors concluded that good sleep quality was linked to sexual activity. Hence, improving sleep quality can be helpful to improve sexual function.

Due to the complexity of FSD, a multifaceted approach, addressing neurobiological, vasoactive, hormonal as well as psychosocial/cultural aspects would be more comprehensive and would address the needs and concerns of the women that suffer from this disorder [24]. Ashwagandha has a member potential to be useful in improving female sexual function due to being an “adaptogen.” which can regulate body metabolic functions in individuals with physical or mental stress [25].

We compared the clinical efficacy and safety of Ashwagandha root extract with an identical placebo in women for improvement of female sexual function. The study product used by us was KSM-66® Ashwagandha capsule (300 mg) administered twice daily orally with water for a period of eight weeks. Ashwagandha has been reported to improve sexual function in women in a randomized, double-blind, placebo-controlled study, where an aqueous extract of Ashwagandha root was used [9]. In this pilot study of 50 patients randomized to AG (n=25) and PL (n=25), there was a greater improvement (reduction) in FSDS scores with AG. In our study, we observed similar findings as reported in this study. The study by Dongre et al. observed improvements in serum testosterone. We did not measure testosterone levels since none of the women participating in our study had any hormonal abnormalities at baseline. We estimated a sample size of 14 required to test a superiority hypothesis that AG is better than PL for improvement in FSFI. However, we included 80 women in our study to have a substantial sample. Our results indicate improvement in female sexual function with both placebo and Ashwagandha. However, there were significantly greater improvements with Ashwagandha over placebo. Thus, these findings suggest a potentially useful role of Ashwagandha in improving sexual health in otherwise healthy women. Ashwagandha roots extract is reported to be safe for human use with a daily dosage of up to 1,000 mg [26,27].

Strengths and limitations of the study

A robust double-blind, placebo-controlled design is the major strength of the study. A major limitation of our study is the well-defined cohort with a homogeneous subject group coming from a specific cross-section of society. Although this design provides greater statistical discrimination power and lower p values for any observed effect size, there is a concern regarding the generalization of these results across the entire population. Future research in a real-world scenario is needed with participants from a wider range of demographics, occupations, and socio-economic statuses. Also, a shorter study duration of eight weeks could prevent any derivations for the long-term benefits.

## Conclusions

This study evaluated the efficacy and safety of Ashwagandha standardized root extract in improving sexual health in otherwise healthy women aged 18 to 50 years having HSDD. Significant improvements in the FSFI scores and FSDS scores were observed with Ashwagandha. Oral administration of standardized Ashwagandha root extract for eight weeks improves female sexual health in otherwise healthy women who do not have any hormonal disturbances.
